# Community-Based Exercise and Lifestyle Program Improves Health Outcomes in Older Adults with Type 2 Diabetes

**DOI:** 10.3390/ijerph18116147

**Published:** 2021-06-07

**Authors:** Morwenna Kirwan, Christine L. Chiu, Mellissa Hay, Thomas Laing

**Affiliations:** 1 Faculty of Medicine, Health & Human Sciences, Macquarie University, North Ryde, NSW 2109, Australia; christine.chiu@mq.edu.au; 2 Diabetes NSW & ACT, Glebe, NSW 2037, Australia; mellissah@diabetesnsw.com.au (M.H.); thomasl@diabetesnsw.com.au (T.L.)

**Keywords:** physical activity, fitness, lifestyle, community based, independent living, exercise physiologist, diabetes

## Abstract

Background: The aim of this study was to assess the efficacy of *Beat It*—a community-based exercise and lifestyle intervention—in improving anthropometric and physical fitness outcomes in older adults with type 2 diabetes mellitus (T2DM). Methods: Australians with T2DM who were aged 60 years or older were included. These individuals were enrolled in *Beat It*, a twice-weekly supervised group exercise and education program conducted over 8 weeks. Anthropometric measurements and physical fitness parameters were assessed at baseline and completion. Physical fitness measures were then compared to validated criterion standards of fitness levels required by older adults to remain physically independent into later life. Results: A total of 588 individuals were included in the study. At baseline, a substantial proportion of the cohort had physical fitness measures that were below the standard for healthy independent living for their gender and age. Significant improvements in waist circumference and physical fitness were observed post program and resulted in an increase in the number of participants who met the standard for healthy independent living. Conclusions: Participation in *Beat It* improved important health outcomes in older adults with T2DM. A longer-term follow-up is needed to determine whether these positive changes were maintained beyond the delivery of the program.

## 1. Introduction

Diabetes is a chronic condition characterised by high blood glucose levels and is one of the fastest-growing and most serious health conditions globally [1]. In Australia, the prevalence of diabetes is highest in older adults over the age of 65 years [2]. Type 2 diabetes (TD2M) accounts for 85–90% of all diabetes cases and is associated with insufficient levels of physical activity [2]. Older adults with T2DM have higher rates of co-morbidities and experience accelerated functional decline [3].

Physical activity is considered a cornerstone in the management of T2DM [4] and refers to any bodily movement produced by skeletal muscle that requires the consumption of energy. Australian guidelines recommend that individuals with T2DM accumulate a minimum of 210 min per week of moderate-intensity physical activity or 125 min per week of vigorous-intensity physical activity [4]. In people with T2DM, participation in regular exercise has been shown to improve glycaemic control, reduce diabetes-related complications, and improve quality of life [5]. Physical fitness is described as the ability to perform physical activity and activities of daily life effectively at various stages within a persons’ lifecycle. Health-related physical fitness is defined as cardiopulmonary fitness, muscle strength, flexibility, and body composition. Physical fitness is a powerful predictor of all-cause mortality and adverse cardiovascular events [6], and associations between low levels of cardiopulmonary fitness and T2DM have been reported [7].

Targeted exercise prescriptions provide significant clinical benefits [8]. However, similar to many adults, individuals with T2DM face numerous barriers in starting and maintaining a physical activity program. These can include time constraints; health problems preventing exercising; inadequate knowledge of how to exercise; fear of adverse health events; and lack of motivation [9]. While these barriers are not unique to people with T2DM, the daily demands of managing a chronic condition add another layer of complexity to overcoming these barriers.

Effective exercise interventions in adults with T2DM have utilised four intervention components that include (1) personalised exercise prescription, (2) supervision by an exercise professional, (3) fitness testing, and (4) group delivery [10]. However, there is limited evidence to show whether these positive outcomes can be replicated when implemented on a larger scale, in community-based settings, across multiple sites, and with multiple exercise professionals delivering the program [11].

*Beat It* is a community-based exercise and lifestyle program established by Diabetes NSW and ACT in 2010 and supported by the National Diabetes Services Scheme (NDSS) since 2015. This program is an example of translational research, the application of research knowledge into practical ‘real-world’ settings [12]. *Beat It* has been running in its current format since 2015 and is an eight-week program that focuses on supporting individuals in improving their diabetes self-management through group exercise and education sessions. The current study aimed to assess whether *Beat It*, a scaled-up exercise intervention, was effective in improving anthropometric, physical fitness, and psychological outcomes in older adults with T2DM.

## 2. Materials and Methods

*Beat It* involves twice-weekly group-based exercise sessions including moderate-intensity aerobic, resistance, flexibility, and balance-based exercises, as well as education sessions on diabetes self-management. Accredited Exercise Physiologists (AEPs) (*n* = 53) delivered the programs at 67 separate locations within New South Wales (NSW) and the Australian Capital Territory (ACT). Participants attended a one-on-one initial consultation with an AEP which involved completing a pre-exercise screening, baseline measurements, fitness testing, and motivational interviewing to establish program goals. Information obtained from this consult was used to devise a personalised exercise program for the participant accounting for physical capabilities, the current level of fitness, as well as co-morbidities or injuries that may be impacted by exercise. Each exercise program included a dynamic warm-up and cool-down, aerobic (e.g., exercise bikes, treadmills, cross-trainers or stepping), resistance (e.g., machine based, free weights, resistance bands, and body weight), balance (e.g., wobble boards, dura discs, standing balance variations), and flexibility exercises (e.g., static stretching of major muscle groups). During the group exercise sessions, participants completed their exercise program under the supervision of an AEP, who was able to advance or regress exercises in accordance with the participant’s progress. These exercise sessions were held in a variety of settings, dependent on location and availability, including public gyms, community halls, and private clinics. All AEPs completed a specialised facilitator training program called ‘Beat It Trainer’ to ensure consistent and effective delivery of the program. This accredited continuing professional development training course consisted of online learning and a one-day practical workshop. To maintain this certification, *Beat It* trainers were also required to complete a refresher course within 2 years of completing the initial training.

Participants were recruited between July 2017 and April 2019 via the NDSS database or through advertising on the Diabetes NSW and ACT website. Prior to commencing, participants were required to provide evidence of medical clearance to exercise from their general practitioner and were then eligible to attend their initial health and fitness assessment with their designated trainer. The number of participants per group session was limited to 15 to enable individuals to receive suitable supervision and support from their AEP. At the completion of the eight weeks, a final health and fitness assessment was undertaken. For inclusion into the current study, participants had to be 60 years of age or over, and have a clinical diagnosis of T2DM. The Macquarie University Human Ethics Committee approved the study, protocol number 5201950887424.

This study employed a pre–post evaluation design where participants completed in-person physical assessment sessions at baseline and at eight weeks after completion of *Beat It*. Sociodemographic variables including gender, date of birth, and residential postcode, were collected. Postcodes were used to determine Socioeconomic Indexes for Areas (SEIFA) and Accessibility and Remoteness Index of Australia (ARIA) [13], as an area-level measure of socioeconomic status and remoteness. Socioeconomic status was assessed using an area-level approach to categorise a participant’s neighbourhood of residence using the Index of Relative Socioeconomic Advantage and Disadvantage (IRSAD) which ranks every region of Australia by relative socioeconomic advantage and disadvantage [14]. IRSAD was dichotomised into top and bottom 50% of deciles, using the base unit of postcode. Participant residential postcodes were imputed into the PoCoG ARIA Lookup Tool [15] and categorised into major cities, inner regional, and outer regional.

Height and weight were used to calculate body mass index (BMI), and BMI was then categorised as healthy, overweight, or obese according to the WHO [16]. Waist circumference was categorised into normal or at risk based on males <94 cm and females <80 cm according to the Royal Australian College of General Practitioners guidelines for preventative activities [17]. Assessment of upper and lower body strength, aerobic capacity, and lower body flexibility were performed using arm curls or medicine ball throw test; 30 s sit-to-stand test; six-minute walk test (6MWT) and the chair sit-and-reach test, respectively. Upper body strength was assessed using either arm curls or the medicine ball throw test. Participants with wrist, elbow, upper limb injuries, or who had recent surgery were excluded from completing these tests. Baseline and post-program fitness measures were dichotomised into below the fitness standard or meeting the fitness standard based on criterion-referenced fitness standards for older adults [18]. This study used a subset of data from a larger operational dataset. Participants with missing data for gender, age, SEIFA information, as well as baseline and/or post-program data for weight, waist circumference, 6MWT (aerobic capacity), and 30 s sit-to-stand test (lower body strength) were excluded from the analysis of the in-person assessments.

DASS-21 is the short-form of Lovibond and Lovibond’s 42-item self-report which measures the negative emotional states of depression, anxiety, and stress [19]. Participants were asked to use a four-point severity/frequency scale (0 = Did not apply to me at all, 1 = Applied to me to some degree, or some of the time, 2 = Applied to me a considerable degree, or a good part of the time, and 3 = Applied to me very much, or most of the time) to rate the extent to which they have experienced each state over the past week [20]. Scores for DASS–Depression, DASS–Anxiety and DASS–Stress were calculated by summing the scores for the relevant items and multiplying by two [19].

Participants were asked to evaluate the efficacy of the program on their knowledge of physical activity and identify the facilitators and barriers to being physically active. Information including country of birth, language spoken at home, and Indigenous status was collected. Participant perspectives around physical activity and diabetes, confidence to incorporate physical activity into diabetes management, and perceived barriers to engaging with physical activity were collected where participants used a five-point Likert scale to indicate to what extent they agreed with statements. Participants were also asked to list any benefits they had gained through engaging in the program and rate how likely they would be to recommend the program. These questions were developed by Diabetes NSW and ACT with the specific intent of evaluating the program and therefore are not validated tools (Figure S1).

Data analysis was performed using SPSS version 24 (SPSS Inc., Chicago, IL, USA). Descriptive statistics: mean, median, and standard deviations (SD) were calculated for continuous variables and frequencies and percentages for categorical variables. The effectiveness of the *Beat It* program on anthropometric and physical fitness assessments was examined using paired T-tests for continuous variables, stratified by gender. For categorical ordinal variables (e.g., Likert scales), the paired Wilcoxon signed-rank test was used to compare pre- and post-program effects. For dichotomous variables (e.g., below or meets criterion standards) the paired sample McNemar test was utilised to compare pre- and post-program effects. A Bonferroni corrected *p* < 0.001 was considered significant to account for multiple testing.

The required sample size was calculated with G*Power (Version 3.1.9.2) [21]. The sample size calculation was approximated with a paired *t*-test, power of 0.80, one-sided α-error 0.05, based on the primary outcome waist circumference effect size 0.15. The effect size used is based on the literature for older (+60 years) overweight adults participating in randomised clinical trial physical activity interventions between 3 and 9 months [22]. In total, for this study 277 individuals were required to detect a change of ≥ 1.5 cm with a standard deviation of 10 cm.

## 3. Results

A total of 588 individuals were included in the study. These individuals were aged 60 years and over, had reported a diagnosis of T2DM, and had participated in the *Beat It* program at one of 67 locations. Of this cohort, 286 (48.6%) were male, and age ranged from 60 to 84 years old with a mean age 69.8 ± 5.6 years. Over one-third (36.1%) of participants were from lower socioeconomic regions, and 47.4% resided outside of major cities. Table 1 summarises this information.

At recruitment, 89% of the cohort had a BMI that placed them in the overweight or obese category, while for 95% of the cohort their waist circumference measures put them at higher risk of chronic disease [18]. The results of the assessment tests at baseline found that a substantial proportion of the cohort performed below what is considered the standard for healthy independent living for their gender and age [18].

Non-significant improvements in weight and resting heart rate were observed post program. Significant improvements in waist circumference, aerobic capacity, strength, flexibility, and balance were observed post program (Table 2) and were associated with improvements in the number of participants who were, at or above, the fitness standards considered appropriate for healthy independent living for older individuals. For upper body strength, there was a 38.7% increase; for lower body strength, 22.3% increase; for aerobic capacity, 17.4% increase; and for flexibility, 12.4% increase in the proportion of participants who were within the healthy range for independent living (Table 3). Improvements were also observed with a significant reduction in depression, anxiety, and stress (DASS) scores within the cohort (Table S1).

All participants were asked to evaluate the efficacy of the program on their knowledge of physical activity, and their confidence in managing their diabetes. Analysis was undertaken on pre- and post-program evaluation questionnaires from 97 participants. Of this group, 44.3% were male, age ranged from 60 to 82 years old, with a mean age 69.6 ± 5.7 years, 74.2% were born in Australia and 97.9% had English as their first language. Within this group, two participants (1.8%) identified as Australian Aboriginal, and 40.2% of participants were from lower socioeconomic areas.

Significant improvements in knowledge around the amount of physical activity required to maintain optimal health were observed, with a 13.2% improvement following completion of the program (57.9% vs. 71.1%). These participants also reported a significant increase in their confidence to undertake exercise (Z = −3.8, *p* < 0.001).

Participants were asked to rate barriers to engaging in regular physical activity. Significant reductions in perceived barriers were observed regarding motivation (−3.8, *p* < 0.001); knowledge to exercise safely (Z = −6.0, *p* < 0.001); and of social support (Z = −3.7, *p* < 0.001). Participants were also asked to record any benefits that they experienced due to participating in the program. Reported benefits included improved blood glucose levels (38%); social support (37%); energy (68%); and increased motivation to exercise (96.2%). Participants were also highly likely to recommend *Beat It* to other people with a net promoter score of 87 (range −100 to 100).

## 4. Discussion

This translational study demonstrates that community-based group exercise and lifestyle programs can achieve important health outcomes in deconditioned older adults with T2DM. This research supports previous smaller-scale studies [23,24,25] that show supervised and tailored exercise programs, combining aerobic, resistance, flexibility, and balance training, significantly improve physical fitness in this demographic. However, this study establishes that a large-scale, short-duration, multi-site intervention, delivered in metropolitan and rural communities, to both high and low socioeconomic participants can achieve improvements in physical fitness.

With an ageing population, there is a need to adopt initiatives that assist in maintaining an individual’s functional fitness, which, in turn, will reduce the risk of falls and enable independent living. Loss of independence is a significant contributor to reduced quality of life. Research suggests that for individuals managing T2DM, their main health goal is to maintain their independence and activities of daily living [26].

In the current study, a substantial proportion of the cohort at baseline had physical fitness measures that were below the standard for healthy independent living for their gender and age. The improvements in aerobic capacity, strength, flexibility, and balance observed in this study demonstrated that *Beat It* can improve physical fitness in older adults with T2DM, who may otherwise have been at risk of losing their independence.

Physical fitness plays a key role in the health of people with T2DM, as increases in physical fitness can predict improvements in cardiovascular risk factors and insulin sensitivity [27]. In this study, the clinical significance of the observed improvements in the physical fitness tests is difficult to interpret due to the paucity of data on the minimal clinically important difference in such measures for people with T2DM [28]. Validated criterion standards that estimate the levels of fitness required by older adults residing in community dwellings to remain physically independent into later life have been developed [18]. These criterion standards were used as a reference point to interpret the findings of this study. At baseline, a high proportion of participants were below the fitness standards required to remain independent in older age. This result was unsurprising as previous research has demonstrated that adults with T2DM are deconditioned, having lower levels of physical fitness and cardiopulmonary fitness when compared to their age-matched diabetes-free counterparts [7]. Without intervention, these individuals are at potential risk of losing their independence, which has serious implications on long-term healthcare costs. Translational studies, such as *Beat It*, have a valuable role in supporting older adults in making lifestyle changes to remain healthy and independent into older age.

On completion of *Beat It*, there was a significant reduction in waist circumference. Waist circumference is a surrogate marker for abdominal visceral fat [29] and reductions in waist circumference and visceral fat are reported to reduce cardiovascular risk and insulin resistance [30]. Significant improvements in each fitness measure and an increase in the number of participants who met the criterion standards for their age and gender across the measures were observed post program [18]. For one-third of participants, the improvements were not enough to meet the established standard for independent living. It could be speculated that for deconditioned older adults with T2DM, a longer intervention duration is necessary to enable them to reach the fitness standards required for healthy independent living. However, the small and significant improvements in physical fitness among participants in this study are still likely to have beneficial outcomes on functional independence, and common co-morbidities, including osteoarthritis, peripheral vascular disease, mobility impairment, depression, and cognitive impairment [8]. Studies measuring functional exercise capacity in healthy adults have reported improvements of 12% in cardiorespiratory fitness following exercise training [31], which is comparable to the improvements in the 6MWT results observed in this study (17% increase in participants who were above the established standard for independent living). Participants who can maintain a schedule of regular physical activity beyond the 8-week program, will likely see further improvements in physical fitness and functional capacity, which is necessary to maintain independence.

A reduction in depression, anxiety, and stress symptoms was observed in a sub-sample of participants post program. T2DM is known to be an ongoing source of stress, and depression and anxiety can intensify diabetes severity, contributing to poor glycaemic control and decreased ability to maintain medical and behavioural regimens [32]. Participation in exercise is known to be an effective therapy in the prevention and management of mental illness and in the promotion of mental health [33] and may explain the improvements in mental health seen. Improvements may also be attributed to the social support provided by the group setting of the sessions [10]. Interacting with peers within the local community who were of similar health status, as well as the encouragement and guidance from the exercise professional may have also been positive contributing factors [10,11].

Other benefits reported by participants include significantly increased confidence in managing their diabetes and significant reductions in self-perceived barriers to physical activity which included motivation, energy, and knowledge of exercise. The delivery of the program to higher and lower socioeconomic populations in both metropolitan and regional areas should be noted as a strength. A limitation of this study is that it employed a pre–post evaluation with no comparison group, a common design for translational community-based programs [34]. This study shows that the *Beat It* program works in practice, although the operational nature of the dataset resulted in some issues with data matching between the in-person assessments and evaluation questionnaires. The pre- and post-survey questions were developed by Diabetes NSW and ACT with the specific intent of evaluating the program and therefore are not yet validated tools. This limits the generalisability of the findings. Information regarding the length of time since a participants’ diabetes diagnosis and insulin dependence was not available. Lastly, this study only evaluated the short-term effectiveness of the program; a longer-term follow-up is needed to ascertain if these positive health outcomes were maintained over time.

## 5. Conclusions

This study revealed that older adults with T2DM can benefit from the eight-week combined exercise and lifestyle *Beat It* program, with improvements observed in physical fitness, mental health, and general approach to diabetes management. The success of this program supports the feasibility of larger-scale community-based supervised group exercise programs in the management of T2DM. A longer-term follow-up is needed to determine whether these positive changes were maintained beyond the delivery of the program.

## Figures and Tables

**Table 1:** Summary of demographic information.

	ALL *n* = 588 (*n* (%))	Males *n* = 286 (*n* (%))	Females *n* = 302 (*n* (%))
**Age Group** (**Years**)			
60–64 years	115 (19.6)	47 (16.4)	68 (22.5)
65–69 years	170 (28.9)	87 (30.4)	83 (27.5)
70–74 years	183 (31.1)	89 (31.1)	94 (31.1)
75–79 years	85 (14.5)	45 (15.7)	40 (13.2)
80–84 years	35 (6.0)	18 (6.3)	17 (5.6)
**SEIFA/IRSAD Index**			
1–5 (least advantaged)	212 (36.1)	113 (39.5)	99 (32.8)
6–10 (most advantaged)	376 (63.9)	173 (60.5)	203 (67.2)
**ARIA class**			
Major cities	309 (52.6)	161 (56.2)	148 (49.0)
Inner regional	159 (27.0)	72 (25.2)	87 (28.8)
Outer regional	120 (20.4)	53 (18.5)	67 (22.2)

**Table 2:** Paired T-test for anthropometric and fitness measure changes from baseline to 8 weeks post program.

	Males				Females			
	*n*	Baseline Mean (SD)	8 Weeks Mean (SD)	*p*-Value	*n*	Baseline Mean (SD)	8 Weeks Mean (SD)	*p*-Value
Weight (kg)	286	93.5 (15.5)	93.3 (15.9)	0.563	302	80.6 (15.9)	80.4 (16.6)	0.458
Waist circ. (cm)	286	110.1 (12.7)	107.7 (12.6)	<0.001	302	105.5 (13.7)	102.5 (13.1)	<0.001
Resting heart rate (bpm)	286	74.3 (12)	74.5 (13.2)	0.716	302	77 (11.2)	77.4 (11.8)	0.465
Chair sit and reach (cm)	266	−9 (12.8)	−4.8 (12.1)	<0.001	275	−4 (11.6)	−0.1 (10.9)	<0.001
30 s Chair stand (#)	286	16.9 (8.3)	20.7 (10)	<0.001	302	15.6 (7.2)	19.4 (8.8)	<0.001
Balance left (s)	276	20.7 (20.1)	26.8 (21.4)	<0.001	284	18.5 (18.3)	24.6 (20.9)	<0.001
Balance right (s)	275	21.2 (20.7)	27.5 (21.8)	<0.001	285	19.4 (19.1)	24.9 (20.1)	<0.001
6 min Walk test (m)	286	452.8 (114.5)	521.3 (104.5)	<0.001	302	409.2 (104.7)	478.9 (105.2)	<0.001
30 s Arm curl (#)	113	16.7 (6.1)	21 (6.4)	<0.001	95	15.5 (5.9)	19.9 (6.5)	<0.001
Med ball throw (m)	81	4.2 (0.9)	4.6 (1)	<0.001	113	2.9 (0.7)	3.1 (0.7)	<0.001

**Table 3:** Classification of participants at baseline and post program for clinical and fitness measures.

	Male			Female		
	Baseline	8 Weeks	*p*-Values	Baseline	8 Weeks	*p*-Values
**BMI**						
Normal (18.5–24.9)	24 (8.4)	25 (8.7)		43 (14.2)	46 (15.2)	
Overweight (25.0–29.9)	123 (43.0)	126 (44.1)		101 (33.4)	100 (33.1)	
Class I obesity (30.0–34.9)	93 (32.5)	98 (34.3)		76 (25.2)	77 (25.5)	
Class II obesity (35.0–39.9)	39 (13.6)	30 (10.5)		56 (18.5)	53 (17.5)	
Class III obesity (≥40)	7 (2.4)	7 (2.4)	0.03	26 (8.6)	26 (8.6)	0.185
**Waist Circumference (cm)** ^**a**^						
Normal range	23 (8.0)	27 (9.4)		7 (2.3)	8 (2.6)	
Risk of chronic disease	263 (92.0)	259 (90.6)	0.100	295 (97.7)	294 (97.4)	0.313
**Chair Sit and Reach (cm)** ^**b**^						
below standard	126 (46.7))	89 (33.0)		127 (45.7)	96 (34.5)	
meets or above standard	144 (53.3)	181 (67.0)	<0.001	151 (54.3)	182 (65.5)	<0.001
**30 s Chair Stand (#)** ^**b**^						
below standard	155 (54.2)	102 (35.7)		162 (536.6)	84 (27.8)	
meets or above standard	131 (45.8)	184 (64.3)	<0.001	140 (46.4)	218 (72.2)	<0.001
**Six Minute Walk Test (m)** ^**b**^						
below standard	260 (90.9)	208 (72.7)		272 (90.1)	222 (73.5)	
meets or above standard	26 (9.1)	78 (27.3)	<0.001	30 (9.9)	80 (26.5)	<0.001
**30 s Arm curl** (***n*** **=** **223**) ^**b,c**^						
below standard	69 (61.1)	29 (25.7)		72 (63.7)	25 (22.1)	
meets or above standard	44 (38.9)	84 (74.3)	<0.001	41 (36.3)	88 (77.9)	<0.001

^a^ Criterion cut-off value for high risk of chronic disease obtained from Royal Australian College of General Practitioners [17]. ^b^ Criterion referenced fitness standards for age and gender obtained from Rikli and Jones [18]. ^c^ Participants with wrist, elbow, upper limb injuries, or who had recent surgery were excluded from this test.

## Data Availability

The data that support the findings of this study are available on request from the corresponding author, Morwenna Kirwan.

## Supplementary Materials

The following are available online at https://www.mdpi.com/article/10.3390/ijerph18116147/s1, Figure S1: Pre- and post-program survey. Table S1: Changes in DASS scores from baseline and post program.
