# Supplementary material for: Community-Based Exercise and Lifestyle Program Improves Health Outcomes in Older Adults with Type 2 Diabetes
Source: Int J Environ Res Public Health. 2021 Jun 7;18(11):6147. doi: 10.3390/ijerph18116147 (PMC8200982; doi:10.3390/ijerph18116147)
Supplement: Supplementary file 1 [file ijerph-18-06147-s001.zip › IJERPH Supp figure 1 - Pre_Post evaluation survey]

Pre-program evaluation
Beat It

Welcome to the **Beat It program.** Before we begin, we would appreciate if you could take ten minutes to complete this questionnaire for research and quality improvement purposes. Your responses will remain confidential.

Today's date (DD/MM/YYYY) DD/MM/YYYY ____________ **Program Location** (suburb) ____________ **State** ____________

Beat It Trainer's Name: ___________________________

Before we begin, please complete the following questions to generate a unique code for you. You will complete these questions on any future Beat It surveys as well. This code allows us to identify changes in your responses over time without revealing your identity.

Date of Birth: (DD/MM/YYYY) D D M M Y Y Y Y

What is your gender? 1 Male 2 Female 3 Other

First 3 letters of your own first name:
(e.g. If Frank, you would write FRA)

---

1. **Postcode where you live** ☐☐☐☐

2. **Do you identify as Aboriginal or Torres Straight Islander?**
   - 1 No
   - 2 Yes, Aboriginal
   - 3 Yes, Torres Straight Islander
   - 4 Both, Aboriginal and Torres Straight Islander
   - 5 Prefer not to disclose

3. **In what country were you born?**
   - 1 Australia
   - 2 Other, please specify________________________

4. **What is the primary language you speak at home?**
   - 1 English
   - 2 Other, please specify________________________

5. **According to the Australian Physical Activity Guidelines, how many minutes of moderate intensity aerobic exercise (e.g. aerobic activities that increase your breathing and heart rate), should adults aim for each DAY to maintain good health?**
   ________________________minutes per day

6. **According to the Australian Physical Activity Guidelines, how many sessions of strength / resistance based exercise should adults aim for each WEEK to maintain good health?** *Select the option you believe is most correct.*

None ☐	1 day per week ☐	*At least* 2 days per week ☐	*At least* 3 days per week ☐	*At least* 4 days per week ☐	5 or more days per week ☐

7. **To what extent do you agree with the following statements:**	Strongly disagree ☹	Somewhat disagree ☹	Neutral 😐	Somewhat agree ☺	Strongly agree ☺
I include planned exercise as a part of my diabetes management.	1	2	3	4	5
I have the confidence to exercise.	1	2	3	4	5
I have a good understanding of how to exercise to improve my diabetes management.	1	2	3	4	5
Overall, I know what lifestyle changes I need to take to manage my diabetes and prevent future problems with my health	1	2	3	4	5
I am confident that I can make changes that are recommended to me to manage my diabetes well.	1	2	3	4	5

8. **Is there anything that limits you from participating in planned exercise on a regular basis? *Please select one answer for each statement***

**To what extent do you agree with the following statements:**	Strongly disagree ☹	Somewhat disagree ☹	Neutral 😐	Somewhat agree ☺	Strongly agree ☺
I lack the motivation to exercise	1	2	3	4	5
I lack the time to exercise	1	2	3	4	5

**To what extent do you agree with the following statements:**	Strongly disagree ☹	Somewhat disagree ☹	Neutral 😐	Somewhat agree ☺	Strongly agree ☺
I do not have the energy to exercise	1	2	3	4	5
I am worried exercise will cause an injury and/or pain	1	2	3	4	5
I do not have access to appropriate resources (e.g. equipment or gym facilities)	1	2	3	4	5
I do not know how to exercise properly/ safely	1	2	3	4	5
I do not have the social support to exercise	1	2	3	4	5
The cost of exercise equipment and/or exercise facilities	1	2	3	4	5

9. Are there any additional barriers to exercise that you experience? If yes, please specify:

Thank you for taking the time to complete this survey, we hope you enjoy the program.
If you have any queries about the program, please contact 1300 136 588 or send an email to beatit@diabetesnsw.com.au.

INFORMATION COLLECTED WITHIN THIS SURVEY IS COVERED BY THE NDSS PRIVACY POLICY. FOR MORE INFORMATION, VISIT HTTPS://WWW.NDSS.COM.AU/PRIVACY-POLICY

Post-program evaluation
Beat It

Thank you for completing the **Beat It program** over the past 8 weeks, we hope you enjoyed it. We would appreciate if you could take a few minutes to complete this questionnaire. Your responses will remain confidential and only be used for quality improvement and research purposes.

Today's date (DD/MM/YYYY) DD/MM/YYYY____________ **Program Location** (suburb) ____________ **State** ____________

Beat It Trainer's Name: ______________________________

Before we begin, please complete the following questions to generate a unique code for you. You will complete these questions on any future Beat It surveys as well. This code allows us to identify changes in your responses over time without revealing your identity.

Date of Birth: (DD/MM/YYYY) [D][D][M][M][Y][Y][Y][Y]

What is your gender? [1] Male [2] Female [3] Other

First 3 letters of your own first name:
(e.g. If Frank, you would write FRA) [ ][ ][ ]

Postcode where you live [ ][ ][ ][ ]

---

1. **According to the Australian Physical Activity Guidelines, how many minutes of moderate intensity aerobic exercise (e.g. aerobic activities that increase your breathing and heart rate), should adults aim for each DAY to maintain good health?**
   ____________________minutes per day

2. **According to the Australian Physical Activity Guidelines, how many sessions of strength / resistance based exercise should adults aim for each WEEK to maintain good health?**
   Select the option you believe is most correct.

None	1 day per week	*At least* 2 days per week	*At least* 3 days per week	*At least* 4 days per week	5 or more days per week
☐	☐	☐	☐	☐	☐

3. **To what extent do you agree with the following statements:**	Strongly disagree ☹	Somewhat disagree ☹	Neutral 😐	Somewhat agree ☺	Strongly agree ☺
I include planned exercise as a part of my diabetes management.	1	2	3	4	5

To what extent do you agree with the following statements:	Strongly disagree ☹	Somewhat disagree ☹	Neutral 😐	Somewhat agree ☺	Strongly agree ☺
I have the confidence to exercise.	1	2	3	4	5
I have a good understanding of how to exercise to improve my diabetes management.	1	2	3	4	5
Overall, I know what lifestyle changes I need to take to manage my diabetes and prevent future problems with my health	1	2	3	4	5
I am confident that I can make changes that are recommended to me to manage my diabetes well.	1	2	3	4	5

4. **Following completion of the Beat It program, is there anything that limits you from participating in planned exercise on a regular basis? *Please select one answer for each statement***

To what extent do you agree with the following statements:	Strongly disagree ☹	Somewhat disagree ☹	Neutral 😐	Somewhat agree ☺	Strongly agree ☺
I lack the motivation to exercise	1	2	3	4	5
I lack the time to exercise	1	2	3	4	5
I do not have the energy to exercise	1	2	3	4	5
I am worried exercise will cause an injury and/or pain	1	2	3	4	5
I do not have access to appropriate resources (e.g. equipment or gym facilities)	1	2	3	4	5
I do not know how to exercise properly/ safely	1	2	3	4	5
I do not have the social support to exercise	1	2	3	4	5
The cost of exercise equipment and/or exercise facilities	1	2	3	4	5

5. Are there any additional barriers to exercise that you experience? If yes, please specify:

6. What benefits do you feel that you have gained from participating in the Beat It program? Tick all that apply

- ☐ My blood **glucose** levels are better
- ☐ My blood **pressure** medication has been reduced
- ☐ My doctor took me off all **blood pressure medication**
- ☐ My **diabetes medication** has been reduced
- ☐ My doctor took me off all **diabetes medication**
- ☐ Improved social support network/ companionship
- ☐ Improved aerobic fitness levels
- ☐ Improved balance
- ☐ Improved flexibility
- ☐ Improved strength
- ☐ Higher energy levels
- ☐ Reduced pain
- ☐ None, I didn't get any benefits from the Beat It program

7. If you experienced other benefits not listed, please describe them below

8. How do you think the program could be improved?

9. How likely is it that you would recommend this program to others? *(0 = not at all likely to 10 = extremely likely)*

0 1 2 3 4 5 6 7 8 9 10

NOT AT ALL LIKELY — EXTREMELY LIKELY

10. What is the most important reason for your score?

11. Is there any other feedback you would like to provide about the Beat It program?

Thank you for your time.

If you have any queries about the program, please contact 1300 136 588 or send an email to beatit@diabetesnsw.com.au.

INFORMATION COLLECTED WITHIN THIS SURVEY IS COVERED BY THE NDSS PRIVACY POLICY. FOR MORE INFORMATION, VISIT HTTPS://WWW.NDSS.COM.AU/PRIVACY-POLICY
