# Supplementary material for: Community-Based Exercise and Lifestyle Program Improves Health Outcomes in Older Adults with Type 2 Diabetes
Source: Int J Environ Res Public Health. 2021 Jun 7;18(11):6147. doi: 10.3390/ijerph18116147 (PMC8200982; doi:10.3390/ijerph18116147)
Supplement: Supplementary file 1 [file ijerph-18-06147-s001.zip › IJERPH Supp table1.pdf]

Supplementary Table 1. Changes in DASS scores from baseline and post-program

	n	Baseline<br><br>mean (SD)	8 weeks<br><br>mean (SD)	Pre - Post<br><br>mean (95% CI)	p value
**Depression**	86	6.3 (7.1)	4.4 (5.4)	1.9 (0.7 – 3.0)	0.003
**Anxiety**	86	4.8 (5.6)	3.8 (5.1)	1.0 (0.1 – 1.9)	0.026
**Stress**	86	8.3 (7.0)	6.2 (5.6)	2.1 (1.0 – 3.3)	0.001
